# Assessment of choroidal osteoma complicating choroidal neovascularization by optical coherence tomography angiography

**DOI:** 10.1007/s10792-017-0503-9

**Published:** 2017-03-31

**Authors:** Ceying Shen, Shu Yan, Min Du, Hong Zhao, Ling Shao, Yibo Hu

**Affiliations:** Department of Zhengzhou Second People Hospital, Ophthalmology, Zhengzhou Eye Hospital, Zhengzhou Ophthalmic Institution, Zhengzhou Hanghai Middle Road No. 90, Zhengzhou, 450000 China

**Keywords:** Choroid osteoma, Choroidal neovascularization, Optical coherence tomography angiography, Fundus fluorescent angiography

## Abstract

**Purpose:**

Choroidal osteoma (CO) frequently leads to progressive visual loss due to complications of secondary choroidal neovascularization (CNV).We report herein the function of optical coherence tomography angiography (OCTA) in observation of CO complicating CNV.

**Methods:**

A 25-year-old female presented to our hospital with chief complaint of sudden unilateral visual acuity decrease for one week, with metamorphopsia in the left eye. Her best corrected visual acuity was 0.12 in the left eye. Then complete ophthalmological examinations including fundus photography, B-scan ultrasound, fundus fluorescent angiography, and spectral-domain optical coherence tomography (SD-OCT) were performed. She was diagnosed as CO on the basis of these results. But the diagnosis could not explain the sudden visual loss and submacular hyperreflective lesion by SD-OCT. Furthermore, she underwent OCTA and indocyanine green angiography.

**Results:**

A diagnosis of classic juxtafoveal CNV secondary to CO was made eventually. Then she was treated with an intravitreous injection of ranibizumab twice. The visual acuity got better and better during the treatment, and the efficacy was stable, giving rise to both subjective and anatomic improvement.

**Conclusions:**

Optical coherence tomography angiography has the advantage of varying the segmentation and scrolling through the different retinal layers, and layer-specific observation of blood flow in each layer. In addition, OCTA can measure the vessel area change of CNV and provide a better appreciation of CNV, observing the efficacy more elaboratively and quantizedly. OCTA makes promising noninvasive identification of the CO-related CNV.

## Background

Choroidal osteoma (CO) is a benign choroidal tumor which contains mature bone [1]. It is most present in young healthy females in the second decade of life [2]. Although most cases are asymptomatic, metamorphopsia, blurred vision, and visual field defects may be initial symptoms [3, 4].The prognosis of the tumor is deteriorated by the presence of choroidal neovascularization (CNV). In this study, we report herein a case of CO complicating CNV that was specifically diagnosed by optical coherence tomography angiography (OCTA).

## Case presentation

**Fig. 1:**
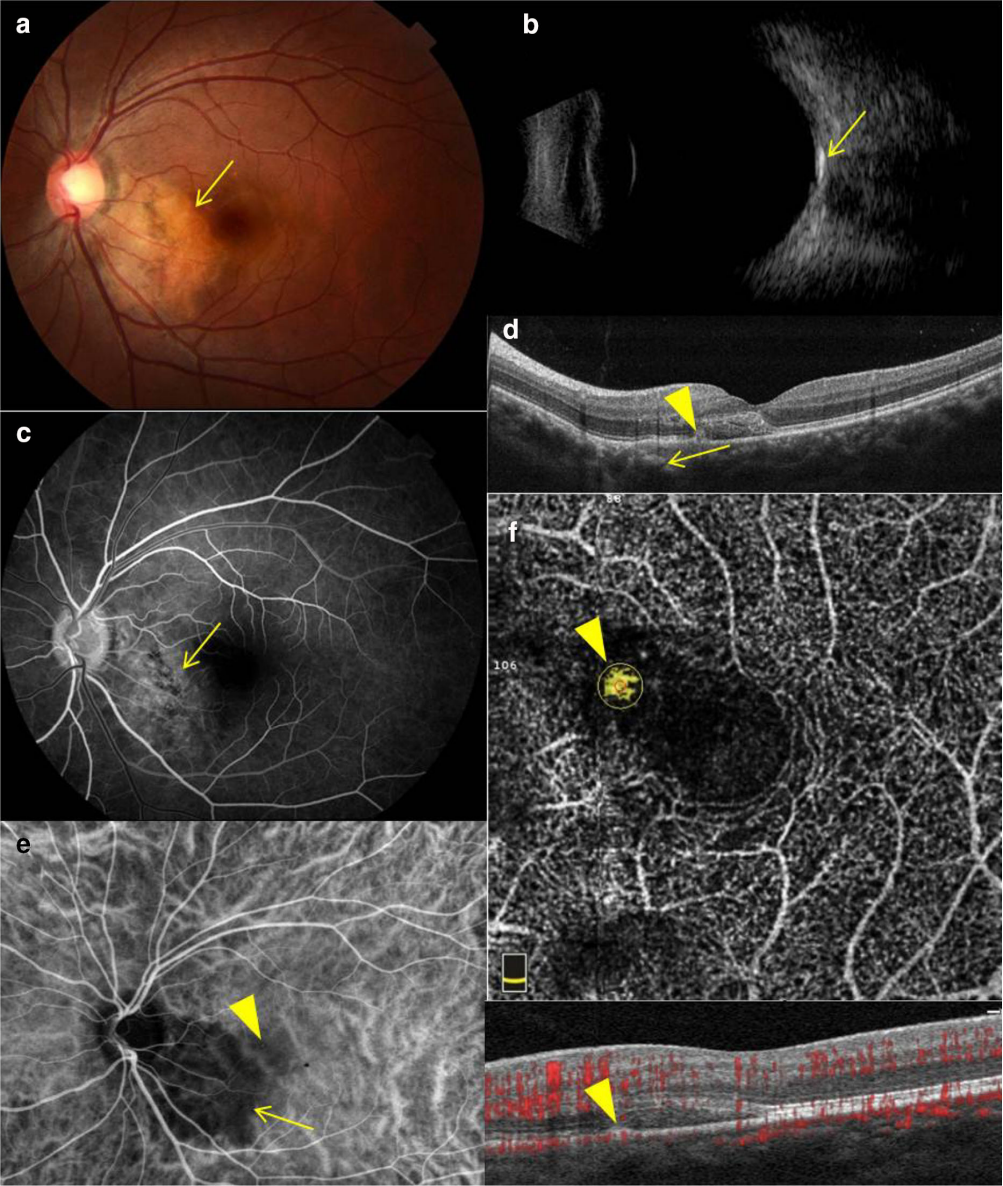
Ophthalmological examinations of a 25-year-old female with choroid osteoma complicating choroidal neovascularization. Fundus photography **a** showed peripapillary subretinal *orange* and *yellow* mass (*yellow arrow*), without involvement of fovea, but accompanied with submacular blood. B-scan ultrasound **b** delineated a high reflective echo at the posterior pole with shadowing of the retrobulbar tissues. FFA **c** demonstrated peripapillary mottled fluorescence (*yellow arrow*), and macular hypofluorescence, without late staining indicating the absence of neovascularization. SD-OCT **d** revealed submacular hyperreflectivity, bulge of RPE (*yellow arrowhead*), and nasal choroidal hyperreflectivity (*yellow arrow*). ICGA **e** showed there was a *dark area* at the peripapillary with faint hyperfluorescence at the macular (*yellow arrowhead*). OCTA images of 3 mm × 3 mm, **f** exhibited that there was abnormal irregular vascular signal in the outer retina layer and the choriocapillaris layer (*yellow arrowhead*), and peripapillary hyperreflectivity was present in the choriocapillaris layer. The osteoma showed no flow in the choriocapillaris layer. There was vascular morphology in the RPE bulge on the B-scan OCTA images corresponding to the abnormal vascular signal in the angiography

A 25-year-old female presented to our hospital with chief complaint of sudden unilateral visual acuity decrease for one week, with metamorphopsia in the left eye. Her best corrected visual acuity was 1.0 in the right eye and 0.12 in the left eye. Intraocular pressure was 14 mmHg in the right eye and 16 mmHg in the left eye. The grade of relative afferent pupil defect (RAPD) was 3+ in the left eye. There was no other relevant past ocular, medical or family history. She underwent complete ophthalmological examinations including fundus photography, B-scan ultrasound, fundus fluorescent angiography (FFA), and spectral-domain optical coherence tomography (SD-OCT). Fundus photography showed peripapillary subretinal orange and yellow mass, without involvement of fovea, but accompanied with submacular blood (Fig. 1a). B-scan ultrasound delineated a high reflective echo at the posterior pole with shadowing of the retrobulbar tissues (Fig. 1b). FFA demonstrated peripapillary mottled fluorescence, and macular hypofluorescence, without late staining indicating the absence of neovascularization (Fig. 1c). SD-OCT revealed submacular hyperreflective lesion, bulge of retinal pigment epithelium (RPE), and nasal choroidal hyperreflectivity (Fig. 1d). She was diagnosed as CO on the basis of these results. But the results could not explain the sudden visual loss and submacular hyperreflective lesion. In addition, to further confirm, she underwent indocyanine green angiography (ICGA) and OCTA. ICGA showed there was a dark area at the peripapillary with faint hyperfluorescence at the macular, indicating the presence of CNV (Fig. 1e).

The instrument used in the OCTA was to obtain amplitude-decorrelated angiography images (software version: 2015.1.0.90; Optovue, Inc., Fremont, CA, USA). OCTA (3 mm × 3 mm) exhibited that there was abnormal irregular vascular signal in the outer retina layer and the choriocapillaris layer, and peripapillary hyperreflectivity was present in the choriocapillaris layer. The osteoma showed no flow in the choriocapillaris layer. There was vascular morphology in the RPE bulge on the B-scan OCTA images corresponding to the abnormal vascular signal in the angiography (Fig. 1f). A diagnosis of CO complicating CNV was confirmed eventually. CNV detected in the region was responsible for the subretinal fluid. And the flow area of CNV was 0.024 mm^2^ visible on OCTA. Then she was treated with an intravitreous injection of 0.5 mg ranibizumab (Lucentis; Genentech, South San Francisco, CA). The risks and benefits of the intravitreal injection of ranibizumab were explained to the patient, and written consent was obtained.

**Fig. 2:**
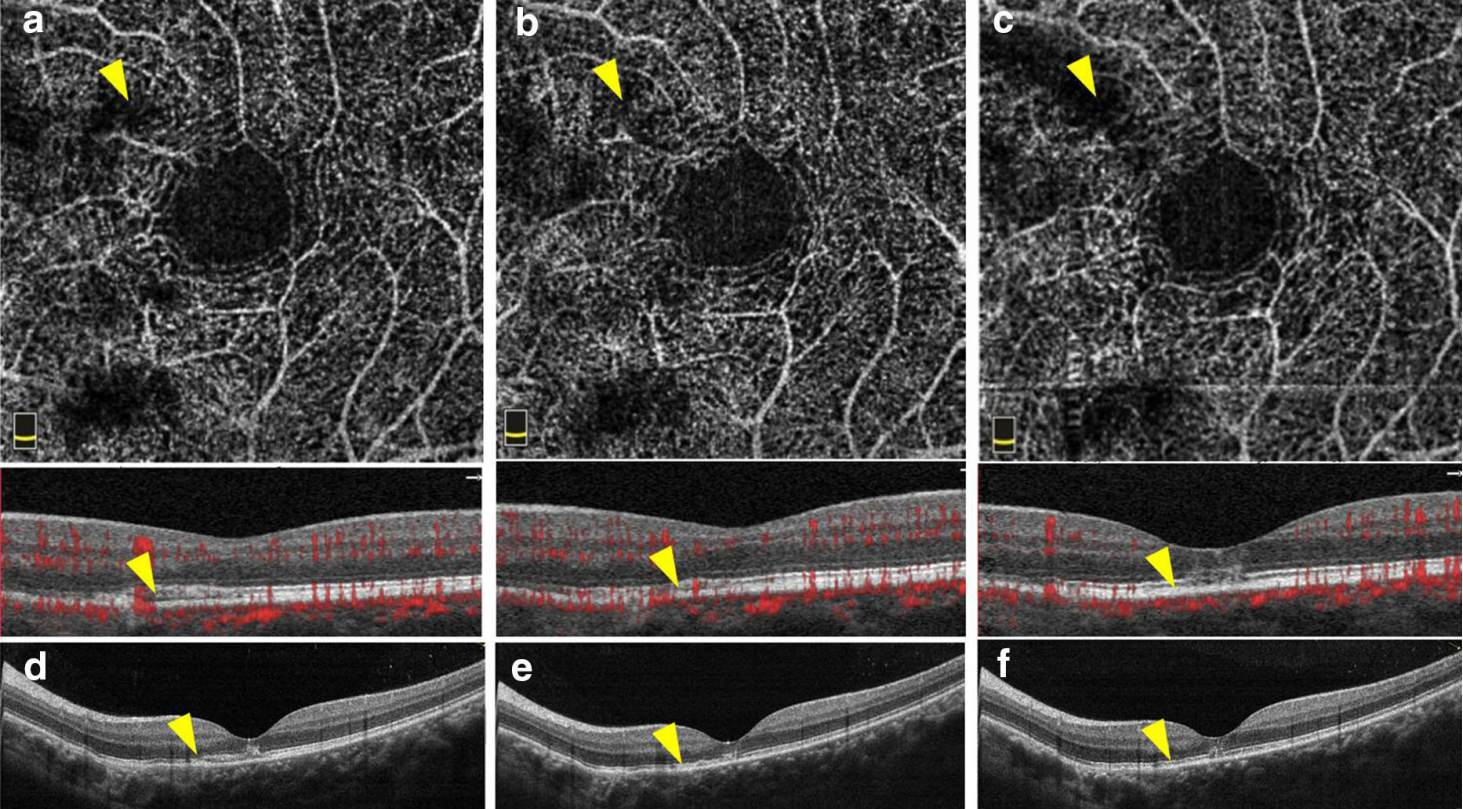
Optical coherence tomography angiography and SD-OCT for the patient during the follow-up. Two weeks after intravitreous injection, abnormal vascular signal was absent in the outer retina layer and the choriocapillaris layer (*yellow arrowhead*) by using OCTA. **a** SD-OCT, **d** revealed that submacular hyperreflectivity decreased, bulge of RPE disappeared (*yellow arrowhead*), but nasal choroidal hyperreflectivity sustained. One month after operation, OCTA showed that abnormal vascular signal was absent in the outer retina layer and the choriocapillaris layer (*yellow arrowhead*). **b** SD-OCT, **e** revealed the absence of submacular hyperreflectivity, and discontinuous ellipsoid zone and interdigitation zone, without bulge of RPE. One month after the second injection, abnormal vascular signal was not detected (*yellow arrowhead*) by OCTA. **c** SD-OCT, **f** revealed that discontinuous ellipsoid zone and interdigitation zone were in regression, without bulge of RPE (*yellow arrowhead*)

Two weeks after intravitreous injection, she came back to recheck, and the visual acuity got improved. Her best corrected visual acuity increased to 0.3, with a reduction of metamorphopsia in the left eye. SD-OCT revealed that submacular hyperreflectivity decreased, bulge of RPE disappeared, but nasal choroidal hyperreflectivity sustained. Abnormal vascular signal was absent in the outer retina layer and the choriocapillaris layer by using OCTA. This patient was sensitive to ranibizumab. The surgery yielded favorable outcomes. One month after operation, her best corrected visual acuity was 0.3 in the left eye. SD-OCT revealed the absence of submacular hyperreflectivity and discontinuous ellipsoid zone and interdigitation zone, without bulge of RPE. OCTA showed that abnormal vascular signal was absent in the outer retina layer and the choriocapillaris layer. She was then performed the second intravitreous injection of 0.5 mg ranibizumab. One month after the second injection, the patient complained of an evident reduction of metamorphopsia and the best corrected visual acuity increased to 0.4 in the left eye. SD-OCT revealed that discontinuous ellipsoid zone and interdigitation zone were in regression, without bulge of RPE. Abnormal vascular signal was not detected by OCTA. The visual acuity got better and better during the treatment, and the efficacy was stable, giving rise to both subjective and anatomic improvement (Fig. 2).

## Discussion

In 1978 Gass et al. first described the disease of CO in four healthy young women with characteristic ophthalmoscopy appearance of a slightly elevated, juxta-papillary, yellowish orange, choroidal lesion with well-defined margins [5]. CO frequently causes progressive visual decline as a result of pigment epithelial decompensation and atrophy of the fovea, or as a result of complications of secondary CNV [6]. CNV develops in 47% of CO patients by 10 years and 56% by 20 years, and it is a major cause of visual impairment [2]. When serous retinal detachment is complicated by CO, it is a recognized association that triggers a search for possible underlying subretinal neovascularization [3]. If it was not detected by FFA or OCT, ICGA should be performed to exclude the subretinal neovascularization. Now we have had a new noninvasive diagnosis examination to preclude CNV, which is OCTA.

Optical coherence tomography angiography provides a high-resolution image of the microvasculature of the retina at various levels in the retina and choroid such as superficial capillary plexus, deep capillary plexus, outer retina, and choriocapillaris in the para- and peri-foveal areas [7, 8]. The advantage of varying the segmentation and scrolling through the different retinal layers, and layer-specific observation of blood flow in each layer makes OCTA widely used in clinical. In our patient, the secondary CNV was not detected during the follow-up. So the change of vessel area was not observed. We set the layer to the choriocapillaris level and then operated the moving of the scan line to detect CNV rigorously. CNV was detected with OCTA in areas where it was undetectable on the FFA due to the diffuse hyperfluorescence. Of note, there have been publications describing CNV secondary to CO detected by OCTA [9, 10]. In our study, the CNV showed regression of lesion size after treatment, same as Szelog et al. reported [9]. Otherwise, we measured the vessel area change of CNV, observing the efficacy more elaboratively and quantizedly. Above all, the detection of CNV secondary to CO is facilitated with OCTA, as the neovascularization could be covered by osteoma in dye-based angiography.

The common choroidal tumors misdiagnosed as CO include choroidal hemangiomas, choroidal melanoma, and choroidal metastases. Choroidal haemangioma is a benign vascular tumor, orange in color and classically located in the macular or paramacular region of the eye [11]. Associated clinical features are exudative retinal detachment, macular edema, pigmentary changes within the RPE, subretinal fibrosis over the surface of the lesion, and orange pigment [12]. Ultrasonography helps to differentiate choroidal hemangiomas from other choroidal tumors [12]. Choroidal melanoma is suggestive of melanoma such as greater tumor thickness, the presence of subretinal fluid and overlying orange pigment, tumor margin near the optic disk, and the absence of drusen and halo [13]. OCT generally shows gentle domed shaped, smooth surface topography with relatively fresh subretinal fluid demonstrating shaggy photoreceptors [14]. Choroidal metastases appear as a yellow mass deep to the retina, often with overlying subretinal fluid [15].

## Conclusions

In the present study, we demonstrate OCTA findings in patients with CO complicating CNV. It is necessary to perform noninvasive examinations during the follow-up to observe the apostasis, replacing the use of FFA or ICGA. Meanwhile, OCTA can detect the area change of CNV and provide a better appreciation of CNV. OCTA may serve as a marker for patients at a risk for CNV secondary to CO. And it makes promising noninvasive identification of the CO-related CNV. Further study with more number of patients is still warranted.

## Abbreviations

CO: Choroidal osteoma
CNV: Choroidal neovascularization
OCTA: Optical coherence tomography angiography
FFA: Fundus fluorescent angiography
SD-OCT: Spectral-domain optical coherence tomography
RAPD: Relative afferent pupil defect
ICGA: Indocyanine green angiography
RPE: Retinal pigment epithelium
